# Decoding plant thermosensors: mechanism of temperature perception and stress adaption

**DOI:** 10.3389/fpls.2025.1560204

**Published:** 2025-03-25

**Authors:** Tongdan Zhu, Xi Cheng, Chengwen Li, Ye Li, Changtian Pan, Gang Lu

**Affiliations:** ^1^ Department of Horticulture, College of Agriculture and Biotechnology, Zhejiang University, Hangzhou, China; ^2^ Bio-breeding Center, Zhejiang Seed Inductry Group Xinchuang Bio-breeding Co., Ltd., Hangzhou, China; ^3^ Department of Agronomy, Heilongjiang Agricultural Engineering Vocational College, Harbin, China; ^4^ Key Laboratory of Horticultural Plant Growth, Development and Quality Improvement, Ministry of Agricultural, Zhejiang University, Hangzhou, China

**Keywords:** thermosensor, temperature, stress, plants, crops

## Abstract

Global climate change, characterized by increased frequency and intensity of extreme temperature events, poses significant challenges to plant survival and crop productivity. While considerable research has elucidated plant responses to temperature stress, the molecular mechanisms, particularly those involved in temperature sensing, remain incompletely understood. Thermosensors in plants play a crucial role in translating temperature signals into cellular responses, initiating the downstream signaling cascades that govern adaptive processes. This review highlights recent advances in the identification and classification of plant thermosensors, exploring their physiological roles and the biochemical mechanisms by which they sense temperature changes. We also address the challenges in thermosensor discovery and discuss emerging strategies to uncover novel thermosensory mechanisms, with implications for improving plant resilience to temperature stress in the face of a rapidly changing climate.

## 1 Introduction

Plants, as sessile organisms, are constantly exposed to a range of environmental stress, including heat, cold, salinity and drought. These abiotic stresses are major limiting crop growth, productivity, and quality, contributing significantly to global food insecurity (He et al., 2018; Sivaji, 2021). With the escalating effects of climate change, heat stress has emerged as one of the most pressing challenges to agricultural productivity (Janni et al., 2020). For instance, studies predict that wheat production could decrease by 6% for each degree Celsius increase in global temperature (Asseng et al., 2015; Zaveri and D, 2019). Moreover, in crops like cereals, peas, lentils, and chickpeas, even brief episodes of heat stress (above 24°C) during the reproductive phase can negatively impact floral fertility, while sustained temperatures of 35°C or higher can result in total crop failure. The impact of heat stress on crop yield varies across species and geographic regions, with reductions ranging from 40% to 85% (Janni et al., 2020).

Unpredicted variability in temperature is also associated with frequent extreme low-temperature events. There is evidence that the Arctic region has warmed more than twice as fast as the global average, a phenomenon referred to as Arctic amplification (Cohen et al., 2014). This accelerated warming has led to significant reductions in Arctic sea ice and spring snow cover, which coincides with a period of ostensibly more frequent extreme weather events across the Northern Hemisphere mid-latitudes, including severe winters (Cohen et al., 2014). Low temperatures pose a widespread environmental stress that significantly inhibits agricultural productivity worldwide by impeding plant growth and development (Mishra et al., 2019). For example, temperatures below 15°C can impair soybean’s growth and development, leading to potential yield loss (Gass et al., 1996). Cold stress also damages flag leaves and spikes, affects the grain number per spike and grain filling rate, leading to a substantial reduction in final wheat production (Fuller et al., 2007; Thakur et al., 2010). A research investigation carried out by the International Rice Research Institute demonstrated a 10% reduction in rice grain yields per 1°C rise in the minimum temperature during the dry season’s growing period (Peng et al., 2004). Given these challenges, understanding the genetic, molecular, and physiological components involved in temperature stress sensing and response is critical for advancing plant biology and improving crop resilience.

Plants have developed intricate mechanisms to perceive environmental temperature signals, regulate growth and reproduction, and even store temperature memory to optimize agronomic traits (Ding and Yang, 2022). Numerous studies have explored plant temperature sensing, responses, and associated signaling pathways. In particular, the identity of plant thermosensors located at the start of the signaling pathways has long been sought (Noguchi and Kodama, 2022). As early as the year 2000, Suzuki and colleagues identified the *cyanobacterium* histidine kinase 33 (Hik33) is the thermosensor that regulates desaturase gene expression in response to temperature downshifts (Suzuki et al., 2000). The identification of plant thermosensors has gained significant attention since the discovery of CHILLING TOLERANCE DIVERGENCE 1 (COLD1) in 2015 (Ma et al., 2015). COLD1 has been identified as a key player in sensing cold temperatures, as demonstrated by Ma et al. (2015). The study suggests that COLD1 may initiate cold signaling through its physical interaction with RGA1, leading to Ca^2+^ influx into the cytoplasm, which triggers downstream responses to chilling stress. In contrast, phytochrome B (phyB) functions as a thermosensor, primarily sensing warm temperatures (Jung et al., 2016; Legris et al., 2016). phyB undergoes conformational changes in sensing temperature fluctuations, typically within the range of 15–30°C, thereby regulating plant growth and development in accordance with environmental conditions (Jung et al., 2016; Legris et al., 2016). While significant progress has been made in identifying and characterizing plant thermosensors like COLD1 and phyB, it is important to clarify the precise roles of these proteins to avoid misinterpretation of their functions. However, the term “thermosensor” is occasionally misused. A typical example is H2A.Z, which plays roles in both transcriptional activation and repression. Although H2A.Z is rapidly displaced from nucleosomes in response to elevated temperature (Kumar and Wigge, 2010; Cortijo et al., 2017), this displacement is not a direct thermosensing mechanism. Cortijo et al. (2017) demonstrated that H2A.Z eviction does not occur at high temperatures *in vitro* or in reconstituted nucleosome systems. Instead, the rapid eviction of H2A.Z at the +1 position under high-temperature conditions depends on the recruitment of heat shock factor A1a (HSFA1a) and potentially other HSFA1 transcription factors to heat shock elements near transcription start sites (Cortijo et al., 2017). Additionally, the deacetylation of histone H3, regulated by POWERDRESS (PWR) and HISTONE DEACETYLASE 9 (HDA9), regulates genes that are also modulated by H2A.Z dynamics, suggesting a potential role for these factors in regulating H2A.Z deposition in plants (Tasset et al., 2018). Together, these findings highlight the need to study how upstream factors such as HSFA1a, PWR, and HDA9 are regulated by temperature, in order to better understand the thermosensing mechanisms that control H2A.Z’s response to temperature changes. To promote more rigorous research on plant thermosensors, several criteria have been proposed (Vu et al., 2019): (1) temperature fluctuations directly alter the activity or conformation of the sensor, (2) these changes are crucial for the sensor to interpret and transmit the temperature stimulus, and (3) the sensor’s ability to detect temperature has a direct effect on the plant’s temperature responses.

From a thermodynamic standpoint, temperature changes can disrupt the equilibrium of biological systems (Reynolds et al., 1992). In nature, temperature fluctuations can have profound effects on living cells by altering the properties and activities of biomolecules such as nucleic acids, proteins, and lipids. Many organisms, including plants, have evolved thermosensors that exploit these properties. In microorganisms and animals, thermosensors are generally classified into four categories based on their chemical nature: DNA, RNA, protein, and plasma membrane-associated protein-based sensors (Sengupta and Garrity, 2013). DNA and RNA thermosensors sense temperature changes through alterations in their secondary and tertiary structures. For example, DNA can bend at low temperatures, and melting of this bent structure promotes transcription as temperature rises (Falconi et al., 1998). In *Shigella*, the DNA bend of the *virF* gene relaxes, releasing the histone-like nucleoid-structuring repressor and enabling transcription factors to bind, thereby activating *virF* gene transcription at temperatures above 32°C (Falconi et al., 1998). Similarly, RNA molecules may form stem-loop structures that inhibit translation, which is disrupted at higher temperatures, allowing translation to proceed (Cortijo et al., 2017; Chung et al., 2023). Protein and membrane-associated protein thermosensors undergo conformational changes or alterations in activity in response to temperature shifts (D. Zhang et al., 2019; Chung et al., 2023). In *B. subtilis*, the membrane-associated histidine kinase DesK, which senses temperature-dependent changes in membrane thickness and activates a pathway to restore membrane fluidity at low temperature by regulating the expression of genes involved in membrane adaptation (Inda et al., 2016).

So far, three major types of thermosensors in plants have been identified: RNA-based thermosensors, protein-based thermosensors, and plasma membrane-associated protein-based thermosensors, including photoreceptors, Ca^2+^-permeable channels, and other proteins. This review summarizes recent findings on plant thermosensors, drawing comparisons with thermosensors in other organisms. Understanding the molecular mechanisms underlying plant temperature sensing and response will provide critical insights for the genetic engineering of temperature-stress-tolerant crops, helping mitigate the impact of climate change on global crop yields.

## 2 RNA thermosensors

Ribonucleic acid (RNA) is a single-stranded nucleic acid molecule and made up of ribonucleotides. RNA polymerase synthesizes RNA from DNA that is functional for protein-coding (messenger RNA, mRNA) or non-coding RNA genes. Because of these functions, RNA molecules are of the following types: mRNA, rRNA, tRNA, snRNA, snoRNA, miRNA, and lncRNA (Cech and Steitz, 2014). RNA molecules are capable of adopting secondary and tertiary structures that are highly sensitive to temperature, allowing them to function as thermosensors. These structures, which are energetically favorable at specific temperatures, regulate RNA stability, pre-mRNA splicing, and translation efficiency, all of which are influenced by temperature changes (Mizushima et al., 1997). Typically, RNA secondary structures are stabilized at lower temperatures and disrupted at higher temperatures, which in turn affects ribosome binding and translation efficiency (Chełkowska-Pauszek et al., 2021; Georgakopoulos-Soares et al., 2022). Moreover, the 5′-untranslated regions (UTRs) of mRNAs may harbor thermolabile stem-loop structures that exhibit temperature-dependent conformational changes, which in turn modulate translation (Di Martino et al., 2016). Recent studies have demonstrated that lncRNA *FLINC* is down-regulated at higher ambient temperature and affects ambient temperature-mediated flowering in Arabidopsis (Severing et al., 2018). Additionally, *miR156* was highly upregulated, while *squamosa promoter binding protein-like* (*SPL*) was downregulated, which further induced *FLOWERING LOCUST* (*FT*) and *FRUITFULL* expression in Arabidopsis under heat stress (Kim et al., 2012; Stief et al., 2014). Therefore, it is speculated that non-coding RNAs could also be used as a thermoresensor to mediate plant responses to temperature fluctuations.

RNA-based thermosensors offer the advantage of rapid response to temperature fluctuations due to their direct conformational changes, enabling swift modulation of downstream signaling pathways. Previous studies have shown that some RNA thermosensors, which control heat shock and virulence genes, function like molecular zippers, reversibly opening and closing in response to ambient temperature shifts (Falconi et al., 1998). These RNA-based mechanisms have been observed in bacteria, and similar temperature-sensitive regulation is found in eukaryotes. For instance, mRNA 5′-UTR secondary structures are implicated in heat shock responses in *Drosophila* (Coleman-Derr and Zilberman, 2012) and *Trypanosomes* (Kumar and Wigge, 2010). In plants, several RNA thermosensors have been proposed to modulate responses to heat stress and other temperature-related environmental cues (**Table 1**).

**Table 1:** List of various RNA thermosensors discussed in this review.

RNA	Plant species	High temperature treatment threshold	Effects	Phenotypic analysis	References
*PHYTOCHROME-INTERACTING FACTOR 7* (*PIF7*)	Arabidopsis	27–32°C	The hypocotyl and petiole elongation	*pif7* mutants had short hypocotyl and petiole	(Capovilla et al., 2017; Chung et al., 2020)
*Photosystem I P700 chlorophyll a apoprotein A1* (*psaA*)	*Chlamydomonas reinhardtii*	40°C	The protein abundance	Removing the secondary structure sequence of the *psaA* 5′-UTR promoted protein translation at 25°C	(Chung et al., 2020, 2023)
*FLOWERING LOCUS M* (*FLM*)	Arabidopsis	27°C	The thermos-responsive flowering	*FLM-ΔE2* (high level of *FL*M-δ) mutants were late flowering, and *FLM-ΔE3* (high level of *FLM-β*) mutants were early flowering	(Capovilla et al., 2017)
*Heat shock transcription factor A2* (*HsfA2*)	Arabidopsis	42–45°C	The growth and survival	*hsfa2* mutants were unable to maintain acquired heat tolerance	(Liu et al., 2013)
*HsfA6e*	Wheat	36–45°C	The thermotolerance	*tahsfA6e* or *tahsp70* mutants exhibited reduced heat tolerance	(Wen et al., 2023)
*HSFA9s*	*R. tomentosa*	45°C	The thermotolerance	Overexpression *OEHSFA9a* and *OEHSFA9c* could improve plants survived after heat stress	(Wen et al., 2023; Li et al., 2024)

### 2.1 PIF7

**Figure 1:**
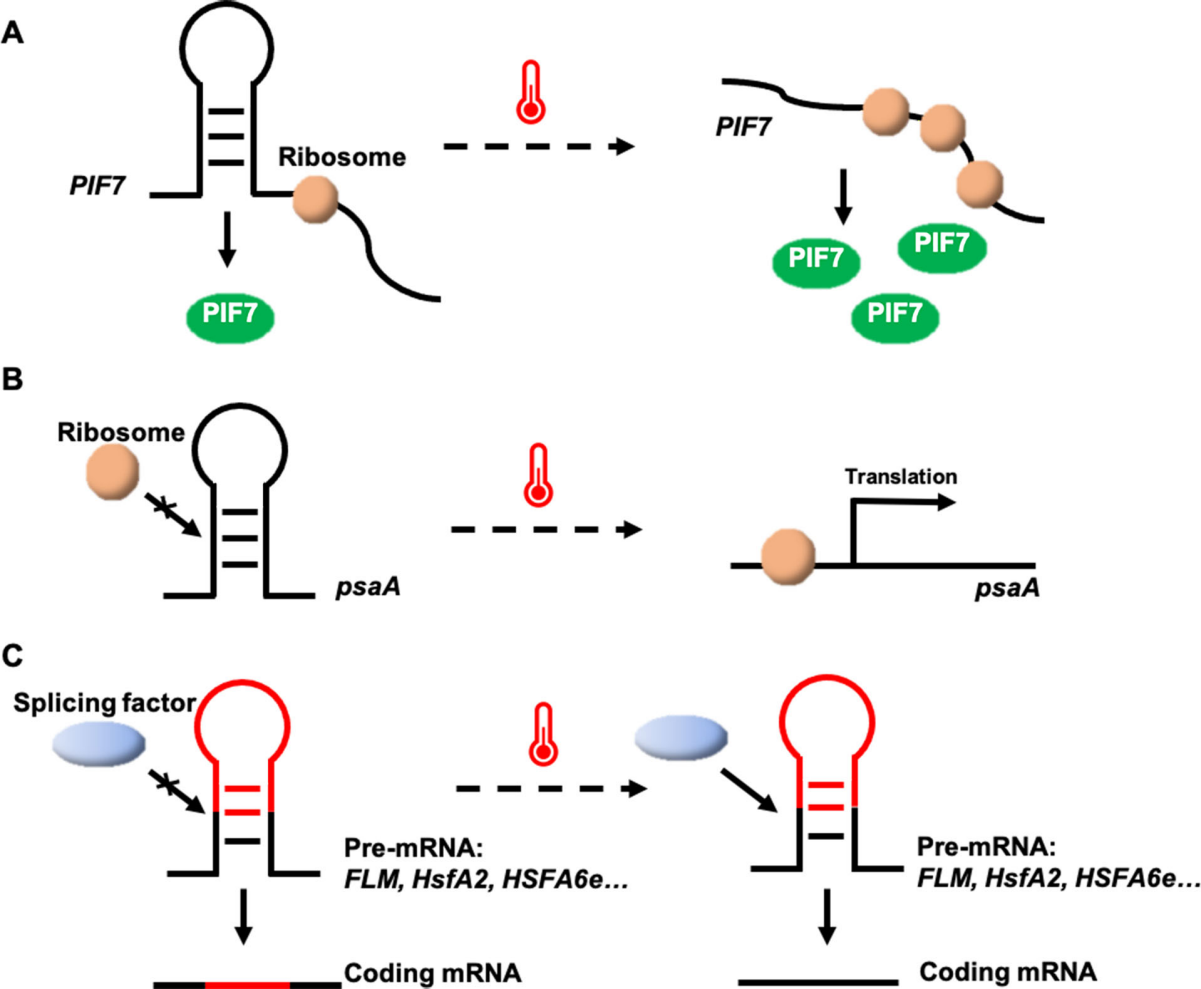
Potential thermosensing mechanisms in RNA thermosensors. **(A)** The heat-sensitive hairpin structure in the *PIF7* mRNA 5′-UTR becomes a looser conformation at high temperatures, enhancing protein translation. **(B)** *psaA* mRNA senses temperature rising to unfold the hairpin structure within its 5′-UTR and facilitates psaA translation. **(C)** Temperature changes may affect alternative splicing by altering RNA secondary structure.

A thermosensor based on secondary structure changes in the *PHYTOCHROME-INTERACTING FACTOR 7* (*PIF7*) mRNA has been identified in Arabidopsis (Chung et al., 2020). A stem-loop structure within the 5′-untranslated region (UTR) of *PIF7* mRNA undergoes conformational shifts in response to temperature. At low temperatures, the AUG-proximal hairpin structure is more compact, while at higher temperatures, the hairpin structure relaxes and partially unfolds, enhancing *PIF7* mRNA translation (**Figure 1A**). This reversible conformational change results in the accumulation of PIF7 proteins, which activate the thermomorphogenesis pathway by inducing the transcription of key genes, such as the auxin biosynthesis gene *YUCCA8*, promoting hypocotyl and petiole elongation. Consequently, *PIF7* mRNA functions as an RNA-based thermosensor that mediates plant responses to temperature fluctuations (Chung et al., 2020).

### 2.2 psaA

Structured RNAs with fundamental sensory and regulatory potential have been discovered in all kingdoms of life (Waldminghaus et al., 2008). RNA thermosensors have been identified not only in plants, but also in algae (K. P. Chung et al., 2023). In the unicellular green alga *Chlamydomonas reinhardtii*, an RNA thermosensor controls translation of the chloroplastic *Photosystem I P700 chlorophyll a apoprotein A1* (*psaA*) mRNA (Chung et al., 2023). The 5′-UTR of *psaA* forms a hairpin-type secondary structure that masks the Shine-Dalgarno sequence (the prokaryotic ribosome-binding site) at 25°C, but melts at elevated temperatures of 40°C, increasing accessibility of the Shine-Dalgarno sequence, initiating and enhancing protein synthesis (**Figure 1B**). It is a valuable tool for inducible transgene expression from the *Chlamydomonas* plastid genome, in that a simple temperature shift of the algal culture can greatly increase recombinant protein yields (Chung et al., 2023). Utilizing the temperature-dependent regulatory mechanism of the 5′-UTR of the *psaA* gene, it is possible to introduce this element into crop genomes through genetic engineering to enhance crop resilience under high-temperature conditions.

### 2.3 FLM

In Arabidopsis, the MADS-box transcription factor gene *FLOWERING LOCUS M* (*FLM*), a key component of the thermosensory flowering time pathway, undergoes temperature-dependent alternative splicing (Posé et al., 2013; Airoldi et al., 2015). *FLM* produces two primary isoforms, *FLM-β* and *FLM-δ*, which differ in the presence of exon 2 or exon 3 (Posé et al., 2013). The overexpression of these variants has opposite effects on flowering, leading to late flowering (overexpression of *FLM-δ*) or early flowering (overexpression of *FLM-β*) (Capovilla et al., 2017). Both FLM-β and FLM-δ proteins compete for interaction with the floral repressor SHORT VEGETATIVE PHASE (SVP) to form an SVP-FLM heterocomplex to regulate the transcription of flowering–related genes, such as *FLOWERING LOCUS T* (*FT*) and *SUPPRESSOR OF OVEREXPRESSION OF CONSTANS1* (*SOC1*) (Posé et al., 2013). *FLM-β* transcript levels decrease and *FLM-δ* transcript levels increase at high temperatures (**Figure 1C**). At low temperatures, the SVP-FLM-β complex predominates, actively repressing flowering. However, at elevated temperatures, the SVP-FLM-δ complex becomes more prevalent, acting as a dominant-negative activator of flowering (Posé et al., 2013; Sureshkumar et al., 2016; Lutz et al., 2017). Although it is not yet known how temperature regulates the alternative splicing of *FLM*, this splicing regulation pathway enables the plant to rapidly sense and respond to fluctuations in ambient temperature, facilitating adaptive flowering time responses.

### 2.4 HsfA2

In Arabidopsis, temperature variations also trigger alternative splicing of the *heat shock transcription factor A2* (*HsfA2*) gene, producing different mRNA isoforms (Liu et al., 2013). *HsfA2* contains only one 324-nucleotide intron, which is able to splice normally to form a full-length protein at 22°C. Under severe heat stress, a 5′-splice site in the *HsfA2* pre-mRNA is activated, leading to the production of *HsfA2-III* (**Figure 1C**), a truncated isoform, S-HsfA2, which binds to the heat shock element (HSE) in the *HsfA2* promoter and activates its own transcription, enhancing heat tolerance (Liu et al., 2013). Similar alternative splicing of *HsfA2* has been observed in tomato. A study comparing wild relatives and domesticated tomato species revealed that efficient pre-mRNA splicing in wild species leads to an accumulation of *HsfA2-II* and suppression of *HsfA2-I*, thereby promoting better adaptation to heat stress (Hu et al., 2020). Further studies have shown that temperature-dependent structural changes at the 3′-splice site of the intron regulate the alternative splicing of *HsfA2* pre-mRNA, suggesting that the secondary structures at this site mediate splicing in response to heat (Broft et al., 2022).

### 2.5 HSFA6e

In wheat, the heat shock transcription factor gene *TaHSFA6e* also undergoes alternative splicing in response to heat stress, generating two major functional transcripts: *TaHSFA6e-II* and *TaHSFA6e-III* (**Figure 1C**) (Wen et al., 2023). TaHSFA6e-III contains a 14-amino acid peptide at its C-terminal (AHA motif), produced by alternative splicing, which enhances the transcriptional activity of three downstream *heat shock protein 70* (*TaHSP70*) genes more effectively than TaHSFA6e-II, improving thermotolerance (Wen et al., 2023). This study highlights a heat-responsive pathway in wheat, where alternative splicing of *TaHSFA6e* regulates the transcriptional efficiency of heat shock proteins, which are localized in stress granules and play a role in translation re-initiation under heat stress conditions (Wen et al., 2023).

Although plants perceive temperature changes through the modulation of alternative splicing of mRNAs (as exemplified by *FLM*, *HsfA2*, and *HSF6e*), the ultimate transduction of temperature signals occurs through the translation of mRNA into proteins.

### 2.6 *HSFA9*s

Current evidence reveals that *HSFA9* belonging to class HSFA acts as an important hub in mediating embryogenesis, germination, photomorphogenesis, and stress protection (Kotak et al., 2007; Zinsmeister et al., 2020; Wang et al., 2024). Recent studies have identified that the *RtHSFA9* genes make crucial contributions to the thermal adaption of *R. tomentosa* by positively regulating the *RtHSFA2a*, *RtHSFA2b*, and *RtHSP* genes (Li et al., 2024). *RtHSFA9a* dramatically enhances plant heat stress tolerance by positively regulating the transcription of *RtHSFA2a*, *RtHSFA2b*, and some *RtHSPs*, such as *RtHSP21.8* and *RtHSP70*. *RtHSFA9b* and *RtHSFA9c* can activate the expression of *RtHSFA2b* and some *RtHSP* genes, consequently taking part in thermal adaption in *R. tomentosa*. In comparison with *RtHSFA9b*, *RtHSFA9c* has higher transcription activity in regulating *RtHSFA2b* and *RtHSP* genes and therefore confers promising thermotolerance to plants (Li et al., 2024). However, the mechanisms by which *RtHSFA9s* perceive accept temperature signals and the subsequent effects of temperature on *RtHSFA9s* remain to be elucidated.

## 3 Protein thermosensors

Proteins, as complex biomolecules composed of thousands of interacting atoms, are inherently sensitive to temperature changes, similar to nucleic acids. Temperature fluctuations can induce significant conformational alterations in proteins, enabling them to regulate various cellular processes, including enzymatic reactions and protein-protein interactions, in response to environmental temperature shifts (Peterson et al., 2007). These temperature-dependent changes in protein structure can also affect the assembly and disassembly of protein complexes, thus modulating cellular functions. Additionally, temperature-induced alterations in protein localization serve as another mechanism through which temperature signals are transduced within the cell (Somero, 1995). In plants, a variety of protein-based and protein-membrane-based thermosensors have been identified, highlighting the importance of protein conformational changes and interactions in plant responses to temperature stress (**Table 2**).

**Table 2:** List of various protein and plasma membrane-associated protein-based thermosensors discussed in this review. .

Protein	Plant species	Temperature treatment threshold	Effects	Phenotypic analysis	References
Phytochrome B (phyB)	Arabidopsis	15–30°C	The hypocotyl elongation	*phyB-9* mutant exhibited reduced heat tolerance	(Jung et al., 2016; Legris et al., 2016; Qiu et al., 2019)
Phototropin (phot)	Liverwort	5°C	Chloroplast distribution	Relocations of the nucleus and peroxisome under cold conditions were impaired in the *MpphotKO* mutant	(Fujii et al., 2017)
EARLY FLOWERING 3 (ELF3)	Arabidopsis	27°C	Thermal control of flowering time	*elf3* mutants were early flowering	(Jung et al., 2020)
Thermo-With ABA-Response 1 (TWA1)	Arabidopsis	45°C	Thermotolerance	*twa1* mutants were conspicuously thermosensitive	(Bohn et al., 2024)
CYCLIC NUCLEOTIDE-GATED Ca^2+^ CHANNELs (CNGCs)	Arabidopsis, moss, and rice	34–45°C; 4°C	Chilling tolerance	Moss *CNGCb* and *Arabidopsis CNGC2/4/6* mutants were more sensitive to heat stress; *OsCNGC9* mutants were more sensitive to chilling stress	(Finka et al., 2012; Gao et al., 2012; Wang et al., 2021)
ANNEXIN1 (ANN1)	Arabidopsis	4°C	Chilling tolerance	The mutant of *AtANN1* and *OST1* substantially impaired freezing tolerance	(Liu et al., 2021)
CHILLING-TOLERANCE DIVERGENCE 1 (COLD1)	Rice	2–4°C	Chilling tolerance	*cold1-1* mutants were chilling sensitive	(Ma et al., 2015)
CRT3-CIPK7	Rice	4°C	Chilling tolerance	*oscrt3-1* mutants were more sensitive to chilling, and the mutation of *OsCIPK7* increased chilling tolerance	(Zhang et al., 2019; Guo et al., 2023)
CHILLING-TOLERANCE DIVERGENCE 6 (COLD6)	Rice	4 ± 0.5°C	Chilling tolerance	*cold6* mutants exhibited higher chilling tolerance, and *osm1* mutants exhibited reduced chilling tolerance	(Luo et al., 2024)
Thermo-tolerance 3 (TT3)	Rice	42°C	Vacuolar degradation	*tt3.1* mutants were more sensitive to heat stress, and *tt3.2* mutants were more heat-tolerance	(Zhang et al., 2022)
Hik33-Hik19	*Cyanobacterium*	22°C	Chilling tolerance	Inactivation of *hik33* and *hik19* reduced the low-temperature-induced accumulation of *desB* and *desD* transcripts	(Suzuki et al., 2000)
Cold-responsive protein kinase 1 (CRPK1)	Arabidopsis	4°C	Chilling tolerance	*crpk1* and *14-3-3κλ* mutants showed enhanced freezing tolerance	(Liu et al., 2017)
Chilling-tolerance in Gengdao/japonica rice1 (COG1)	Rice	4°C	Chilling tolerance	*cog1* mutants were more sensitive to chilling	(Xia et al., 2023)

### 3.1 Phytochrome B

**Figure 2:**
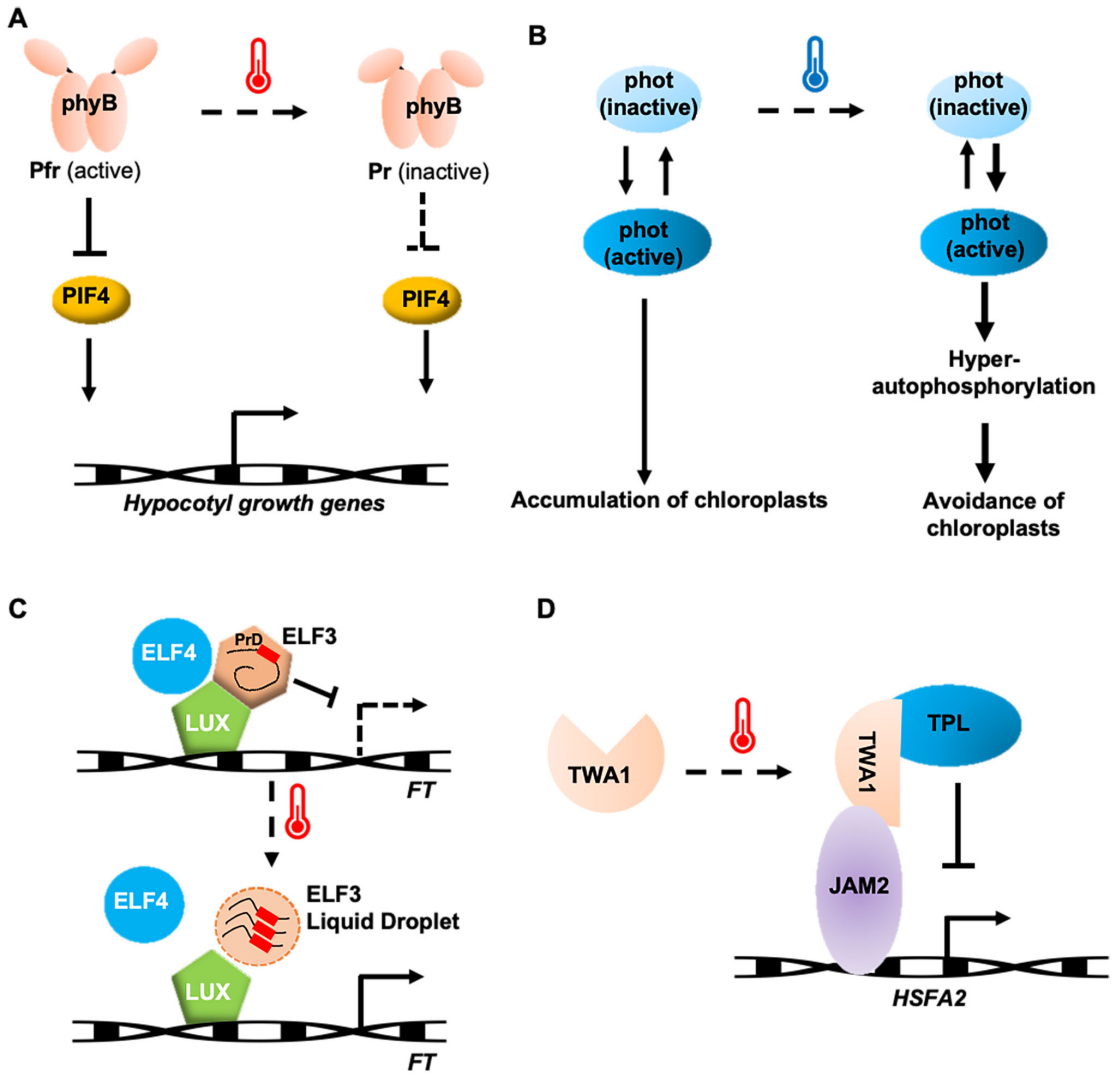
Potential thermosensing mechanisms in protein thermosensors. **(A)** phyB involved in Arabidopsis temperature perception and heat-tolerance formation. **(B)** A phototropin in liverwort functions as thermosensors by preventing the inactivation of its active forms at low temperature. **(C)** ELF3 responds to temperature by phase separation. **(D)** TWA1-mediated transcriptional repression by interacting with JAM2 and TPL. Arrows indicate positive regulation and T-bars indicate negative regulation.

In natural environments, plants sensing temperature changes are closely related to sunlight. Light and temperature in plants are perceived through a common receptor, phytochrome B (phyB) (Jung et al., 2016; Legris et al., 2016). phyB exists in two states: a red-light-absorbing, biologically inactive Pr state, and a far-red-light-absorbing, biologically active Pfr state (Quail et al., 1995; Burgie and Vierstra, 2014). PhyB participates in temperature perception through its temperature-dependent reversion from the active Pfr state to the inactive Pr state. Increased rates of thermal reversion to warmer environments reduce both the abundance of the biologically active Pfr-Pfr dimer pool of phyB and the size of the associated nuclear bodies, which releases the repression of phyB on the *PHYTOCHROME-INTERACTING FACTOR 4* (*PIF4*) (**Figure 2A**). As a result, PIF4 accumulates and promotes hypocotyl elongation by enhancing the expression of auxin biosynthesis genes (Jung et al., 2016; Legris et al., 2016; Qiu et al., 2019). Although there are five phytochrome isoforms (phyA–E) in Arabidopsis, thermal reversion is an order of magnitude faster in phyB than in the others (Burgie et al., 2021). This feature is also found in potato and maize, suggesting that phyB serves as the primary thermosensor among the phytochrome family in many plant species (Burgie et al., 2021; Noguchi and Kodama, 2022).

phyB is also involved in the cold stress response. The key transcription factors mediating cold acclimation, C-REPEAT BINDING FACTORS (CBFs), are rapidly induced by cold stress and bind to *CRT*/*DRE* DNA regulatory elements in the promoters of a subset of *cold-regulated* (*COR*) genes; expression of these *COR* genes renders plants able to tolerate freezing stress (Jaglo-Ottosen et al., 1998; Gilmour et al., 2000). CBFs could interact with PIF3 under cold stress, which attenuates the mutual degradation of the PIF3-phyB complex. Cold-stabilized phyB acts downstream of CBFs to positively regulate freezing tolerance by modulating the expression of stress-responsive genes as well as growth-related genes (Jiang et al., 2020).

### 3.2 Phototropin

Phototropin (phot) is a blue light receptor involved in processes such as phototropism, stomatal opening, and chloroplast repositioning (Christie, 2007), and is the only reported thermal sensor of cold responses in plants to date. Phototropin contains two light-sensitive LOV (Light, Oxygen, or Voltage) domains at the N-terminus and a serine/threonine kinase domain at the C-terminus. The LOV domain exists in both active and inactive forms, determined by whether it combines with FMN (flavin mononucleotide). Under blue light, a covalent cysteine bridge forms between LOV2 and FMN, converting the inactive form into the active form. Upon activation, the phototropin protein undergoes a conformational change, and its kinase domain undergoes subsequent autophosphorylation. Similar to phyB, phototropins also undergo thermal reversion from an active to inactive form, but this process slows down at lower temperatures. This results in the accumulation of the active form of LOV2, increasing autophosphorylation, which determines the accumulation response or cold-avoidance response in liverwort (**Figure 2B**). Phototropin perceives both blue light and temperature, using this information to optimize chloroplast positioning for efficient photosynthesis (Fujii et al., 2017).

Like phototropin and phytochromes in plants, other photoreceptors (e.g., blue-light receptors cryptochromes and UV Resistance Locus 8) also display temperature-dependent lifetimes (Findlay and Jenkins, 2016; Pooam et al., 2021). Therefore, photoreceptors like phyB has already been demonstrated to act as thermosensors (Li and Song, 2024).

### 3.3 ELF3

Temperature changes are part of the plant’s circadian clock perception. The evening complex, consisting of EARLY FLOWERING 3 (ELF3), a large scaffold protein, ELF4, a small α-helical protein, and LUX ARRYTHMO (LUX), a DNA-binding protein, plays a key role in temperature sensing (Box et al., 2015; Nieto et al., 2015). ELF3 contains a PrD domain with a high proportion of polyQ, which varies in length among species and serves as a tunable thermosensor. Increasing the polyQ length enhances thermal responsiveness (Ezer et al., 2017). At 22°C, ELF3 diffuses within the cell and binds to DNA, inhibiting transcription. At 27°C, ELF3 aggregates into a punctate pattern, undergoing phase separation, which prevents ELF3 from binding to flowering genes, thus allowing these genes to be expressed and promoting growth and flowering (**Figure 2C**) (Jung et al., 2020). The temperature sensitivity of ELF3 is modulated by ELF4, suggesting that ELF4 stabilizes ELF3’s function. ELF3 undergoes reversible phase transition through liquid-liquid phase separation (LLPS), making it an ideal temperature sensor (Jung et al., 2020).

### 3.4 TWA1

A recently identified temperature-sensing transcriptional co-regulator in Arabidopsis, THERMO-WITH ABA-RESPONSE 1 (TWA1), is predicted to be an intrinsically disordered protein with a key thermosensory role, functioning through its amino-terminal highly variable region (Bohn et al., 2024). At elevated temperatures, TWA1 accumulates in nuclear subdomains and undergoes conformational changes, allowing it to interact with JASMONATE-ASSOCIATED MYC-LIKE (JAM) transcription factors and TOPLESS (TPL) and TOPLESS-RELATED (TPR) proteins to form repressor complexes (**Figure 2D**). The transcriptional upregulation of *HSFA2* and heat shock proteins depends on TWA1, with orthologues of TWA1 providing different temperature thresholds, consistent with its role in early heat stress signaling (Bohn et al., 2024). Interestingly, specific amino acid changes in the amino-terminal region of TWA1 orthologues are linked to different temperature thresholds. Overexpression of TWA1 could improve crop heat tolerance without negatively affecting plant growth or yield (Bohn et al., 2024), highlighting its potential for application in crop breeding strategies.

### 3.5 Plasma membrane-associated protein-based thermosensors

The plasma membrane, which is one of the most thermally sensitive macromolecular structures in the cell, has long been proposed as a primary candidate for temperature sensing in plants (Balogh et al., 2013; Niu and Xiang, 2018). Subtle changes in temperature can affect various properties of cellular membranes, including fluidity, thickness, permeability, and packing, and thus affect the clustering of important membrane proteins, which are sensitive to ambient conditions (Escribá et al., 2008; Niu and Xiang, 2018). Although membrane lipids lack catalytic ability on their own, changes in their physical state strongly affect the folding, mobility, and activity of integral or membrane-associated proteins (Hayes et al., 2021; Kerbler and Wigge, 2023). To date, several plasma membrane-associated proteins have been proposed as potential thermosensors, including CNGCs, ANN1, COLD1, CRT3-CIPK7, COLD6, TT3, Hik33-Hik19, CRPK1, and COG1 (**Table 2**).

#### 3.5.1 CNGCs

**Figure 3:**
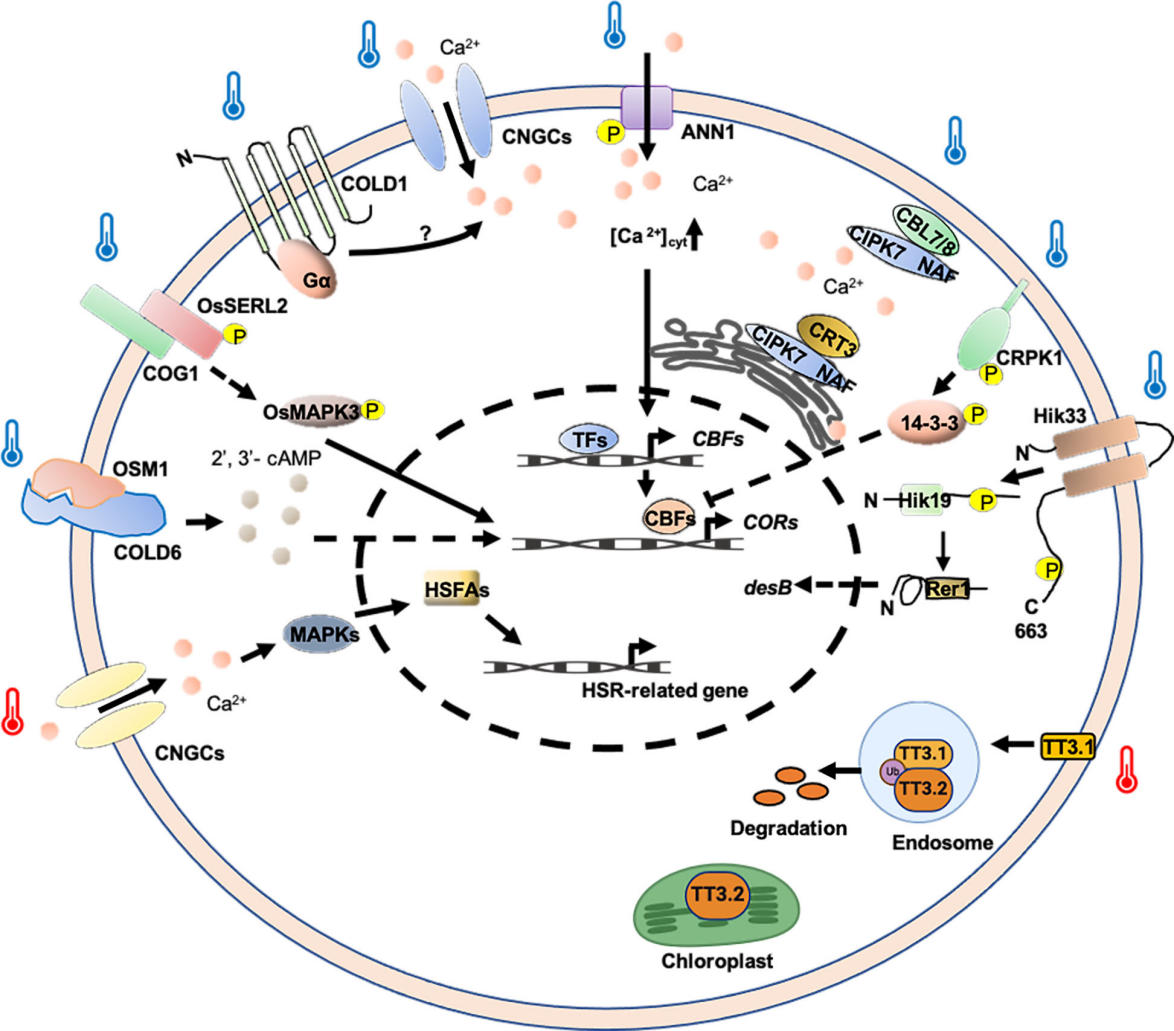
Potential thermosensing mechanisms in plasma membrane-associated protein-based thermosensors. Ca^2+^ and 2′, 3′-cAMP are important molecules involved in regulation temperature-stress response. Cold and heat-induced Ca^2+^ signatures may be decoded by Ca^2+^ sensors or Ca^2+^ -related proteins and thus regulate *COR* and *HSR* gene expression. TT3.1 senses high temperature to translocate to the endosomes, where it degrades protein TT3.2. TT3.2 degradation boosts chloroplast function at high temperatures. COLD6 interacted with cold-induced OSM1 to trigger an increase in the level of 2’, 3’-cAMP to promote chilling tolerance. TT3.1 proteins translocate from the PM to endosomes, ubiquitinating the chloroplast precursor protein TT3.2 to prevent chloroplast thylakoid damage and improving heat tolerance. Hik33 kinase domain might be phosphorylated under cold conditions, and then transferred to Hik19, and finally to Rer1, regulating the expression of the *desB* to adapt to cold stress. CRPK1 was activated by cold stress, phosphorylating 14-3-3 proteins and triggering 14-3-3 proteins to translocate into the nucleus to attenuate the CBF signaling. The COG1-OsSERL2 complex causes the activation of OsMAPK3 to transmit cold signal from the membrane to the cytoplasm, enhancing cold tolerance. Arrows indicate positive regulation and T-bars indicate negative regulation.

Extreme temperature changes can negatively affect membrane fluidity, with cold stress reducing fluidity and heat stress increasing it (Sangwan et al., 2002). Temperature fluctuations are first sensed by plasma membrane CYCLIC NUCLEOTIDE-GATED Ca^2+^ CHANNELs (CNGCs), which encode components of the membrane’s cyclic nucleotide-gated Ca^2+^ channels (Falcone et al., 2004). In plants, CNGCs function in signaling pathways that may be tied to their ability to transport Ca^2+^ rather than other cations into plant cells (Jha et al., 2016). It has been reported that CNGCb gene from *Physcomitrella patens* and its Arabidopsis thaliana ortholog CNGC2/4 act as the primary thermosensors of land plant cells (Finka et al., 2012). Additionally, AtCNGC6 also mediates heat-induced Ca^2+^ influx (Gao et al., 2012). Under heat conditions, the channels quickly open, allowing periplasmic Ca^2+^ to enter and bind calmodulins (CaMs) associated with the cytosolic C-terminal domain of the CNGCs (Niu et al., 2020). Ca^2+^ binding to CNGC-bound CaMs initiates a specific signaling cascade that activates kinases, which then phosphorylate and activate *heat shock transcription factor A* (*HSFA*), promoting the expression of *HSP* genes (**Figure 3**) (Guo et al., 2016; Ohama et al., 2016; Guihur et al., 2022).

In rice, a loss-of-function mutant of *OsCNGC9*, named *cds1* (*cell death and susceptible to blast 1*), is more sensitive to chilling stress (Wang et al., 2021). In this case, OsCNGC9 is phosphorylated and activated by OsSAPK8, a member of sucrose non-fermenting 1-related protein kinases (SnRK) family, triggering Ca^2+^ influx and the activation of cold stress-related genes. Furthermore, OsDREB1A, a rice dehydration-responsive element-binding transcription factor, positively regulates the transcriptional expression of *OsCNGC9*. Thus, OsSAPK8-mediated OsCNGC9 phosphorylation and OsDREB1A-mediated OsCNGC9 expression form a potential positive feedback loop, leading to enhanced OsCNGC9-mediated Ca^2+^ influx, the expression of cold stress-related calcium-dependent protein kinase genes (CPKs) and OsDREB1A, and chilling tolerance in rice (**Figure 3**) (Wang et al., 2021). Additionally, it was shown that rice CNGC14 and CNGC16 are involved in promoting tolerance toward heat and chilling stresses, and are regulators of Ca^2+^ signals in response to temperature stress (Cui et al., 2020).

#### 3.5.2 ANN1

Apart from CNGCs, Annexin 1 (ANN1) also plays a crucial role in increasing cytosolic Ca^2+^ during cold stress. In Arabidopsis, ANN1 mediates cold-triggered Ca^2+^ influx and contributes to freezing tolerance. Under normal conditions, the serine/threonine protein kinase Open Stomata 1 (OST1) interacts with PP2C, which inhibits OST1’s kinase activity. AtANN1 is localized both in the cytosol and at the plasma membrane. Upon cold stress, OST1 is activated and phosphorylates AtANN1. This phosphorylation enhances AtANN1’s Ca^2+^ transport activity and promotes its Ca^2+^-binding activity (**Figure 3**). The dual role of phosphorylation leads to an increase in cold-induced cytosolic Ca^2+^, which indirectly facilitates the expression of CBFs and CORs, positively regulating plant freezing tolerance (Liu et al., 2021). Although these findings provide important insights into the molecular mechanisms of plant responses to cold stress, several questions remain to be addressed. For instance, is the specific mechanism of AtANN1 consistent across different cellular environments? How do the interactions between OST1 and other signaling pathways influence the function of AtANN1? Future research should focus on these aspects.

#### 3.5.3 COLD1

In rice, CHILLING-TOLERANCE DIVERGENCE 1 (COLD1) plays an essential role in cold adaptation by regulating G-protein signaling. COLD1 is localized at the plasma membrane and endoplasmic reticulum (ER) and is involved in sensing cold temperatures, triggering Ca^2+^ signaling for chilling tolerance. A single-nucleotide mutation in COLD1 confers chilling tolerance to japonica rice, originating from the Chinese wild populations of *Oryza rufipogon*. COLD1 interacts with the G-protein α subunit (RGA1), activating Ca^2+^ channels and enhancing G-protein GTPase activity, thereby enhancing stress-related genes (*OsDREB1A*, *OsDREB1B*, *OsDREB1C*, and *OsAP2*) to promote chilling tolerance (**Figure 3**) (Ma et al., 2015). Recently, researchers have further revealed that COLD1 regulates the low temperature tolerance of rice by regulating the metabolism of downstream vitamin E-vitamin K1 (Luo et al., 2021). However, the molecular mechanism of COLD1 sensing low temperature and the Ca^2+^ channels activated by COLD1 remain unclear. In the future, structural biology and other means are needed to further elucidate the principle and conformational changes of low-temperature activation of COLD1.

Additionally, a single-nucleotide polymorphism (SNP2738) in *ZmCOLD1* confers chilling tolerance and is associated with maize adaptation during speciation from teosinte (Zhou et al., 2023). *ZmCOLD1* not only influences the influx of extracellular Ca^2+^, but also the levels of abscisic acid, gibberellic acid, and indole-3-acetic acid, during the germination stage, promoting chilling tolerance under low temperatures (Zhou et al., 2023).

#### 3.5.4 CRT3-CIPK7

In rice, the endoplasmic reticulum (ER)-localized Ca^2+^-binding protein calreticulin (CRT) plays a crucial role in various processes, including Ca^2+^ signaling (Michalak et al., 1999). CRT3 is a plant‐specific family member and differs from the CRT1/2 group that plays a role in SA‐dependent immune responses (Jia et al., 2009; Qiu et al., 2012). The CBL-INTERACTING PROTEIN KINASE (CIPK) is a calmodulin-interacting kinase that positively regulates cold stress responses, potentially through its kinase activity (D. Zhang et al., 2019). Under normal conditions, the interaction between OsCRT3 and OsCIPK7 is characterized by a low binding affinity. The kinase activity of OsCIPK7 is constrained by its intrinsic auto-inhibitory NAF/FISL domain, concomitantly with the maintenance of a diminished cytosolic Ca^2+^ concentration ([Ca^2+^]_cyt_). Under chilling stress, cold signaling induces conformational changes in OsCRT3, enhancing its binding affinity with OsCIPK7 and consequently activating its kinase activity (**Figure 3**). At the same time, OsCRT3, localized to the endoplasmic reticulum (ER), facilitates the increase in cold-induced [Ca^2+^]_cyt_, a signal that is detected by Ca^2+^ sensor proteins OsCBL7/8 (**Figure 3**). These proteins specifically interact with OsCIPK7 on the plasma membrane (PM). This interaction suggests a regulatory mechanism activated by chilling stress in rice, involving OsCRT3, OsCIPK7, Ca^2+^, and its sensor proteins OsCBL7/OsCBL8 (Guo et al., 2023).

#### 3.5.5 COLD6

As previously discussed, secondary messengers serve as critical upstream components in mediating signaling pathways, directly responding to signals from temperature stress sensors. Although it is well-established that temperature sensors initiate Ca^2+^ signaling pathways to mediate cold tolerance in cells, the mechanisms by which sensors interact with other secondary messengers remain poorly understood. Recent studies have identified 2′, 3′-cAMP as a secondary messenger crucial for chilling tolerance in crops (Luo et al., 2024). *CHILLING-TOLERANCE DIVERGENCE 6* (*COLD6*), encoded by a major quantitative trait locus (QTL) gene in rice, interacts with RGA1 at the plasma membrane under normal conditions. Upon exposure to chilling, cold-induced osmotin protein (OSM1) binds to COLD6, displacing RGA1, which triggers the elevation of 2′, 3′-cAMP levels, promoting the expression of cold- responsive genes (**Figure 3**) (Luo et al., 2024). This elevation enhances chilling tolerance, providing a new insight into plant responses to cold environments and offering a potential target for molecular breeding aimed at improving cold tolerance in crops.

#### 3.5.6 TT3

In rice, *Thermo-tolerance 3* (*TT3*) has been identified, which modulates temperature perception based on protein localization and interaction (Zhang et al., 2022). *TT3* is a quantitative trait locus (QTL) comprising two genes: *TT3.1* and *TT3.2*. TT3.1 is a positive regulator of high-temperature stress, acting upstream of TT3.2, while TT3.2 plays a negative role in heat stress response. Under high-temperature stress, the heat stress‐induced plasma membrane (PM)‐localized E3 ligase TT3.1 proteins translocate from the PM to endosomes, where it ubiquitinates the chloroplast precursor protein TT3.2 (**Figure 3**). Subsequently, TT3.2 undergoes vacuolar degradation, preventing chloroplast thylakoid damage and improving heat tolerance (Zhang et al., 2022). However, the mechanism on how TT3.2 accumulation leads to chloroplast damage under heat stress conditions awaits further investigation. Given that the antagonistic effect of TT3.1 on TT3.2 confers protection to chloroplasts under heat stress conditions, overexpression of *TT3.1* or targeted editing/silencing of *TT3.2* could be employed as a strategic approach to enhance thermotolerance in rice.

#### 3.5.7 Hik33-Hik19

Temperature also affects the activity and function of membrane proteins. Evidence suggests that the *cyanobacterium* histidine kinase 33 (Hik33) is the thermosensor that regulates desaturase gene expression in response to temperature downshifts (Suzuki et al., 2000). Hik33 contains 663 amino acid residues and the strongly conserved histidine kinase domain is located near the C-terminus. Hik33 is predicted to span the plasma membrane twice and forms a dimer, whose structure and activity may be influenced by the physical characteristics of lipids in the plasma membrane, such as their fluidity (or the extent of molecular motion), which is controlled by temperature and the extent of unsaturation of the fatty acids (Lau et al., 1997; Yaku and Mizuno, 1997; Singh et al., 1998; Suzuki et al., 2000). When the temperature is decreased (from 34 to 22°C), the histidine residue in the Hik33 kinase domain may be phosphorylated. A phosphate group is then transferred to Hik19, and finally to Rer1, which regulates the expression of the *fatty-acid-desaturase genes B* (*desB*) to adapt to cold stress (**Figure 3**) (Suzuki et al., 2000). Further, Hik33 has been shown to regulate the expression of osmotic stress-inducible genes and also to bind to certain chemicals, indicating that it may function as a multifunctional sensor for a variety of stresses (Jeon et al., 2010).

#### 3.5.8 CRPK1

In addition to algae, there are similar cases in higher plants. In Arabidopsis, cold stress could activate the plasma membrane-localized protein kinase CRPK1 (cold-responsive protein kinase 1), which phosphorylates 14-3-3 proteins in the cytoplasm, thereby triggering 14-3-3 proteins to translocate into the nucleus (Liu et al., 2017). In the nucleus, phosphorylated 14-3-3 proteins form a protein complex with CBF proteins to promote the 26S proteasome-mediated degradation of CBF proteins, thus attenuating the CBF signaling (**Figure 3**). These results reveal that CRPK1 and 14-3-3 proteins act upstream of CBF proteins to negatively regulate plant freezing tolerance (Liu et al., 2017). Additional investigations are required to explore whether manipulating CRPK1 or 14-3-3 proteins could improve cold tolerance without compromising other vital physiological processes.

#### 3.5.9 COG1

Xia et al. (2023) characterized Chilling-tolerance in *Gengdao*/*japonica rice 1* (*COG1*) as a major gene identified in a QTL for positive regulation of chilling tolerance in *japonica* rice, which encodes a cold-induced LRR-RLP located in the plasma membrane and ER. Mechanistic studies have found that COG1 targets and activates the kinase somatic embryogenesis receptor kinase-like 2 (OsSERL2) in a cold-induced manner, promoting chilling tolerance (**Figure 3**). Furthermore, the cold signal transmitted by COG1-OsSERL2 activates OsMAPK3 in the cytoplasm, ultimately inducing a cold-tolerance response (Xia et al., 2023). This study not only advances our understanding of the molecular mechanisms underlying cold stress responses in rice but also highlights the potential of COG1 as a target for breeding cold-tolerant crop varieties. However, further research is needed to explore the broader applicability of these findings across diverse rice cultivars and environmental conditions, as well as to investigate potential trade-offs between chilling tolerance and other agronomic traits.

## 4 Conclusion and prospects

Earth is undergoing an unprecedented period of global climate change, characterized by frequent extreme temperature events. Dramatic temperature fluctuations induced by climate change inhibit plant growth, posing a significant threat to crop production and the sustainability of agricultural systems and food security. In response to this challenge, a range of plant thermosensors have been identified, shedding light on how plants sense and respond to temperature fluctuations (Ding and Yang, 2022; Noguchi and Kodama, 2022). RNA-based thermosensors have been identified frequently. For instance, temperature changes have the capacity to modulate not only the RNA structure, exemplified by *PIF7* and *psaA*, but also influence the alternative splicing of mRNAs, including but not limited to *FLM*, *HsfA2*, and *HsfA6e*. These events triggered by extreme temperature play a pivotal role in temperature and regulating the expression of genes implicated in downstream temperature stress responses.

In contrast to RNA-based thermosensors, protein-based thermosensors have also been extensively characterized in plants, playing a crucial role in mediating temperature perception and signaling. As photoreceptors, phyB and phot undergo thermal reversion to relay downstream signaling perception of temperature change (Jung et al., 2016; Legris et al., 2016; Fujii et al., 2017). ELF3 and TWA1 sense temperature fluctuation through liquid-liquid phase separation (Fujii et al., 2017; Bohn et al., 2024). The fluidity and permeability of cellular phospholipid membranes are altered by extreme temperature changes in plants (Sangwan et al., 2002), suggesting that membrane-localized channels or receptors might sense temperature stress signals. The rapid increases in [Ca^2+^]_cyt_ concentration triggered by extreme temperature are important in mediating downstream temperature stress-related gene expression (Wu et al., 2022; Li et al., 2023). Ca^2+^ signaling-related proteins, such as CNGCs, ANN1, COLD1, and CRT3-CIPK7, play important roles in temperature stress response by regulating Ca^2+^ level. In addition to the concentration of [Ca^2+^]_cyt_, temperature also affects the concentration of 2′, 3′-cAMP (Luo et al., 2024). Consequently, signaling proteins associated with 2′, 3′-cAMP, such as COLD, fulfill critical functions in the sensing temperature stress in plants. Under heat stress, TT3 modulates temperature perception based on protein localization and interaction (Zhang et al., 2022). The lower temperature affects the function of the membrane-located thermosensors. As the temperature decrease, the function of Hik33, CRPK1, and COG1 are activated, thereby transmitting the temperature signal to downstream components (Suzuki et al., 2000; Liu et al., 2017; Xia et al., 2023).

Notably, the interplay between different thermoreceptors, such as *PIF7* and phyB in Arabidopsis, has provided critical insights into the molecular mechanisms of temperature perception. *PIF7*, an RNA thermosensor, is regulated by a hairpin structure within its 5′-UTR. Upon exposure to high temperatures, this structure partially unfolds, enabling translation initiation and leading to an increase in PIF7 protein levels. Concurrently, high temperatures promote the reversion of phyB from its active Pfr state to its inactive Pr state, a process known as thermal reversion. This allows PIF4 and PIF7 to activate thermomorphogenesis genes, demonstrating how plants integrate multiple thermosensors to regulate growth and development.

To cope with unfavorable changes in climate and an increasing global population, it is crucial to cultivate productive, climate-resilient crops. Advances in gene discovery have enabled the use of genetic modification techniques to enhance crop traits (Ding and Yang, 2022). Improving cold tolerance in rice is crucial for minimizing yield loss (Dong et al., 2025). As mentioned earlier, *COLD1* and *COG1* overexpression lines (Ma et al., 2015; Xia et al., 2023), and *cold6* mutants exhibited higher chilling tolerance (Luo et al., 2024), which suggests that the potential of either genetic or transgenic approaches to improve chilling tolerance for rice breeding. Besides, the overexpression of *TT3.1* or knockout of *TT3.2* lines were more heat-tolerant and increased yield by more than 2.5 times (Zhang et al., 2022). Interestingly, TT3.1 and TT3.2 are conserved in other major crops such as maize and wheat (Li and Liu, 2022), which would be meaningful to know whether these orthologous genes could be used for breeding heat tolerant crops. From the discussion, one may conclude that there is great potential to apply the identified plant thermosensors to improve crop temperature stress tolerance and thus increase yields. Further, it is worth exploring how to efficiently apply the thermosensors identified in model plants to crop improvement. The present understanding of individual molecular components in temperature responses may be established. However, the interactive and coordinated mechanisms among multiple molecules remain unclear. Such molecular coordination may play crucial roles in various biological processes. Therefore, further research is needed to understand how multiple molecules coordinate temperature responses during the same stress or developmental processes.

Currently, few DNA thermosensors have been characterized, and most reported thermosensors are RNA, protein, and plasma membrane-associated protein-based thermosensors in plants. RNA can sense temperature changes through changes in secondary structure, while proteins can regulate their function through heat-induced conformational changes. Comparatively speaking, the structural changes of DNA are limited by its double helix conformation, and the regulatory mode is relatively limited (Singh et al., 2022). The potential of DNA as a temperature sensor has not been fully explored, possibly also due to a lack of relevant experimental tools or methods. While DNA-based thermosensors are less commonly discussed, recent studies have begun to uncover their potential roles in plant responses to temperature changes. For instance, temperature-induced changes in DNA methylation patterns and chromatin remodeling have been implicated in regulating gene expression under thermal stress (Song et al., 2021; Talarico et al., 2024). Thus, the potential role of epigenetic mechanisms—such as DNA methylation, RNA methylation, chromatin structure modifications, loss of imprinting, and non-coding RNAs—in temperature sensing remains largely unexplored. Understanding the full spectrum of temperature perception pathways is crucial for improving plant growth and development, and identifying additional thermosensors could play a pivotal role in enhancing crop stress resistance (Raza et al., 2024).

Genetic redundancy and lethality present challenges in identifying new thermosensors. Nevertheless, molecular genetic approaches, coupled with innovative bioimaging techniques such as Ca^2+^ imaging-based forward genetic screens and fluorescence-based Ca^2+^ indicators, are helping to uncover new temperature sensors (Sun et al., 2021; Jin et al., 2024). Moreover, the integration of protein-protein interaction studies, multi-omics techniques (including genomics, proteomics, metabolomics, lipidomics, glycomics, and transcriptomics), bioinformatics, and advanced microscopy is expected to accelerate the discovery of novel thermoreceptors. The diversity of plant responses to temperature anticipates that many new thermosensors and eventually novel sensing mechanisms will be uncovered soon (Casal et al., 2024). Ultimately, translating these findings into practical applications for improving crop resilience to temperature stress through breeding and cultivation techniques will be critical to sustaining global food security in the face of climate change.
